# Platelet behaviour on von Willebrand Factor changes in pregnancy: Consequences of haemodilution and intrinsic changes in platelet function

**DOI:** 10.1038/s41598-017-06959-6

**Published:** 2017-07-25

**Authors:** Jonathan Cowman, Sieglinde Müllers, Eimear Dunne, Adam Ralph, Antonio J. Ricco, Fergal. D. Malone, Dermot Kenny

**Affiliations:** 1 Molecular and Cellular Therapeutics, Royal College of Surgeons in Ireland, Dublin, Ireland; 2 Obstetrics and Gynecology, Royal College of Surgeons in Ireland, Dublin, Ireland; 3 Irish Centre for High-end Computing, National University, Ireland Galway, Ireland

## Abstract

Platelet function in pregnancy is poorly understood. Previous studies of platelet function in pregnancy have used non-physiological assays of platelet function with conflicting results. This study using a physiological assay of platelet function investigated platelet interactions with von Willebrand Factor (VWF) in blood from healthy pregnant women and healthy non-pregnant controls. Blood samples (200 µl) from third-trimester pregnancies (*n* = 21) and non-pregnant controls (*n* = 21) were perfused through custom-made parallel-plate flow chambers coated with VWF under arterial shear (1,500 s^−1^). Multi-parameter measurements of platelet interactions with the immobilized VWF surface were recorded by digital-image microscopy and analysed using custom-designed platelet-tracking software. Platelet interactions with VWF decreased in healthy third-trimester pregnant participants relative to controls. This effect is most likely due to haemodilution which occurs physiologically during pregnancy. Interestingly, platelets in blood from pregnant participants translocated more slowly on VWF under arterial-shear conditions. These decreases in platelet translocation speed were independent of haemodilution, suggesting intrinsic changes in platelet function with pregnancy.

## Introduction

During pregnancy, physiological changes occur such that platelet count falls slightly, together with a drop in haemoglobin concentration and haematocrit (HCT) as blood volume expands, typically by 40%^1^. Platelet function studies in healthy pregnant females compared with healthy non-pregnant female controls have yielded conflicting results, with studies reporting either decreased^2^, increased^3–5^, or no change^6–8^ in either platelet reactivity or platelet activation, as assayed using *ex*-*vivo* methods.

At sites of vascular damage, extracellular matrix proteins such as collagen are exposed to blood. Circulating plasma proteins such as von Willebrand Factor (VWF) bind to the exposed subendothelial matrix and support the recruitment of platelets from the blood stream to form a platelet plug. Platelets initially bind to VWF via the platelet glycoprotein (GP) Ibα receptor^9^. These initial interactions between VWF and the platelet are short-lived, resulting in platelet translocation (stop/start “rolling” motion of the platelet at the blood/surface interface) as GPIbα-VWF bonds are formed and broken^10^. The transient interaction of GPIbα-VWF enables the platelet to slow down adequately to adhere stably to VWF via the platelet integrin GPIIb/IIIa^11^. These initial dynamic platelet interactions on VWF under arterial shear forces are central to any description of platelet function within the circulatory environment.

Previous studies of the effect of pregnancy on platelet function, which examined the response of platelets *ex vivo* to agonists, were limited by the use of non-physiological assays such as light transmission aggregometry or flow cytometry. To address a range of conditions as well as diseases in which platelet function plays a key role, we developed a system capable of measuring platelet function under physiological conditions. We call this system and its results the Dynamic Platelet Function Assay (DPFA). Using novel parallel-plate flow chambers coated with purified human VWF and having internal volumes of a few microliters, in combination with custom-designed platelet-motion-tracking software, the DPFA can accurately measure platelet translocational behaviour on a VWF surface^12, 13^. We have previously demonstrated the capability of this system to detect subtle changes in platelet function by describing age-related changes in platelet behaviour and differences between term and preterm neonates^14, 15^.

The aim of this study was to characterise platelet function in healthy pregnancy. To do this, we compared DPFA-measured platelet function in healthy pregnant females in their third trimester and non-pregnant healthy female controls. Our findings demonstrate that alterations in platelet function that occur in pregnancy are due both to haemodilution and to intrinsic changes in platelet function. To the best of our knowledge, this is the first study of pregnancy to use a multi-parameter dynamic platelet function test to examine platelet-VWF interactions under arterial shear-flow conditions.

## Results

### Study populations

The study enrolled a total of 21 healthy women in their third trimester of a healthy pregnancy and 21 healthy non-pregnant controls. All participants gave informed consent in accordance with the Declaration of Helsinki. Full blood counts were obtained for each donor using a Sysmex KX-21N Hematology Analyzer. Full study population demographics are outlined in Table 1.

**Table 1:** Demographics of healthy non-pregnant controls compared with healthy third trimester pregnancy participants.

Demographics	Healthy nulliparous controls	Healthy third trimester pregnancies
Number	21	21
Age	26 ± 4	31 ± 7
Gestational age at blood draw (weeks)	—	36 ± 4
Platelet count (×10^3^ per μL)	209 ± 55	171 ± 44*
Haematocrit (%)	39 ± 5	31 ± 4***
Gestational age at delivery (weeks)	—	39.6 ± 1.3
Mode of delivery	—	50% VD
Vaginal delivery (VD)		
Elective caesarean section (ELCS)		41% ELCS
Emergency caesarean section (EMCS)		9% EMCS
Birth weight (g)	—	3543 ± 335
Estimated blood loss at delivery (mL)	—	345 ± 213

Data are presented as mean ± SD; *p < 0.05 and ***p < 0.0001.

### Dynamic platelet function on VWF is reduced in healthy pregnancy

**Figure 1:**
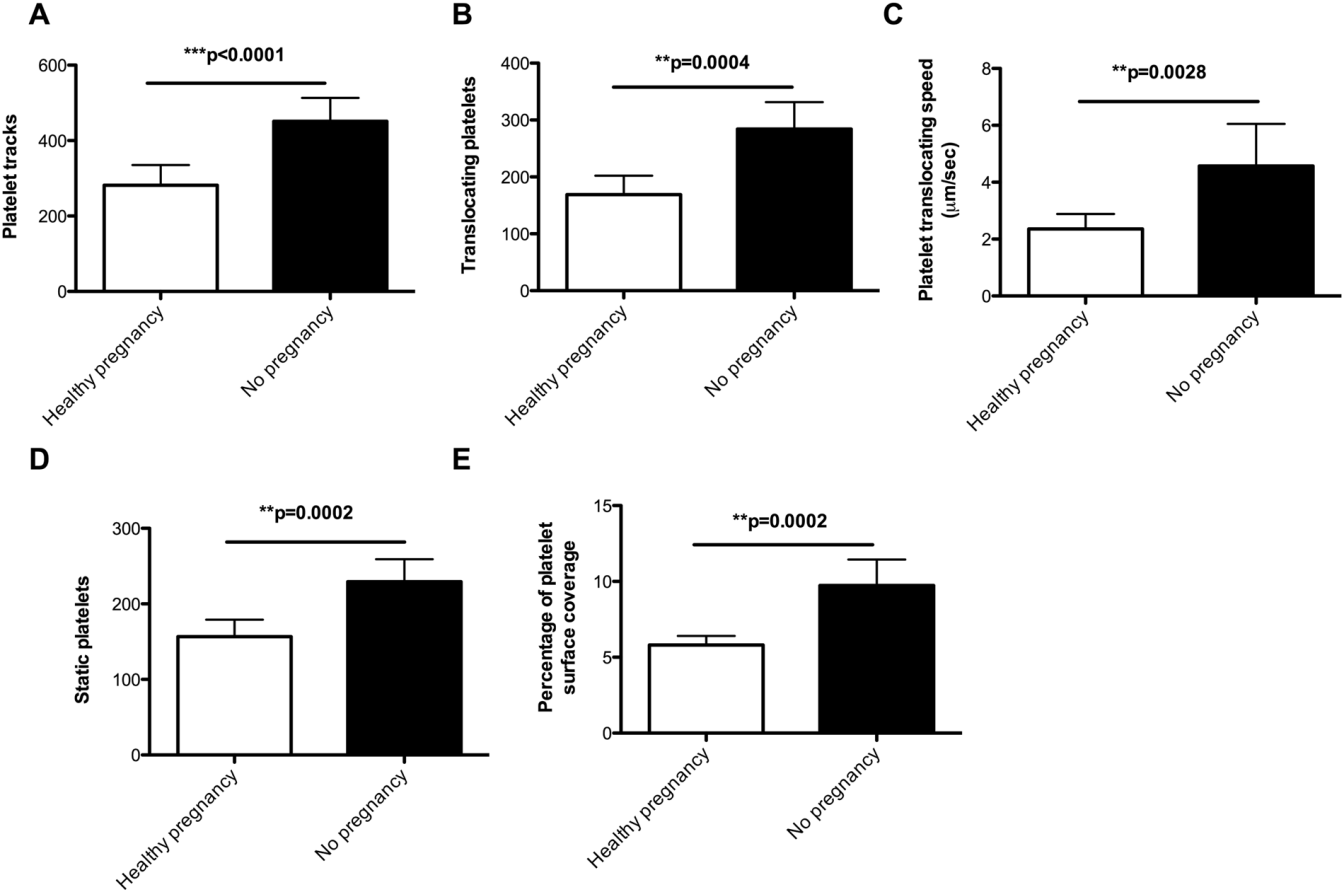
Platelet function on VWF is reduced during pregnancy. Blood from 21 pregnant females in the third trimester of pregnancy and 21 healthy non-pregnant female controls were perfused over VWF at arterial shear rates. Platelet function on VWF was measured using the dynamic platelet function assay. Pregnant females showed a significant reduction in platelet function on VWF (*p < 0.05, **p < 0.01 and ***p < 0.0001). Data are presented as mean ± 95% confidence interval.

The DPFA was used to characterise platelet function in healthy pregnancy (Fig. 1). This assay generates a number of outputs that are closely linked to biological platelet function. The number of translocating platelets is associated with the activity of GPIbα, whereas the number of static platelets is a measure of the engagement of integrin GPIIb/IIIa. Real-time video microscopy tracks the number of fluorescently labelled platelets interacting with the VWF-coated surface of the flow chamber under arterial shear in whole blood. There was a significant reduction in platelet interaction with VWF in the cohort of pregnant females compared to the matched, non-pregnant controls (Table 2).

**Table 2:** Pregnancy induces a dramatic decrease in platelet interactions with VWF.

	Pregnant females (*n* = 21)	Non-pregnant female controls (*n* = 21)	*p* value
Platelet Tracks	281 (227–334)	451 (389–512)	0.0001
Translocating platelets	168 (135–200)	284 (237–330)	0.0004
Translocation Speed μm/sec	2.3 (1.7–2.8)	4.5 (3.0–5.9)	0.0038
Static platelets	156 (133–178)	229 (199–258)	0.0002
% Surface Coverage	5.8 (5.2–6.3)	9.7 (8.0–11.3)	0.0002

Values show mean + 95% CI.

#### The effects of reducing platelet and HCT on dynamic platelet interactions with VWF

Haemodilution is one of the normal physiological changes that occur during pregnancy: the volume of circulating plasma increases, resulting in a lowering of the cell counts. In our subjects, pregnancy was associated with a significant reduction in both platelet count (171 ± 44 × 10^3^ vs. 209 ± 55 × 10^3^ per μL, *p < 0.05) and haematocrit (HCT) (31 ± 4 vs. 39 ± 5% ***p < 0.0001) compared to the non-pregnant controls. In order to determine if the reduction in platelet count and HCT levels within our pregnant cohort were responsible for the differences in dynamic platelet interactions with VWF in our assay, we performed experiments to monitor the effect of *ex*-*vivo* reduction of platelet counts or HCT on DPFA outputs.

#### Reducing platelet count reduces dynamic platelet interactions with VWF

**Figure 2:**
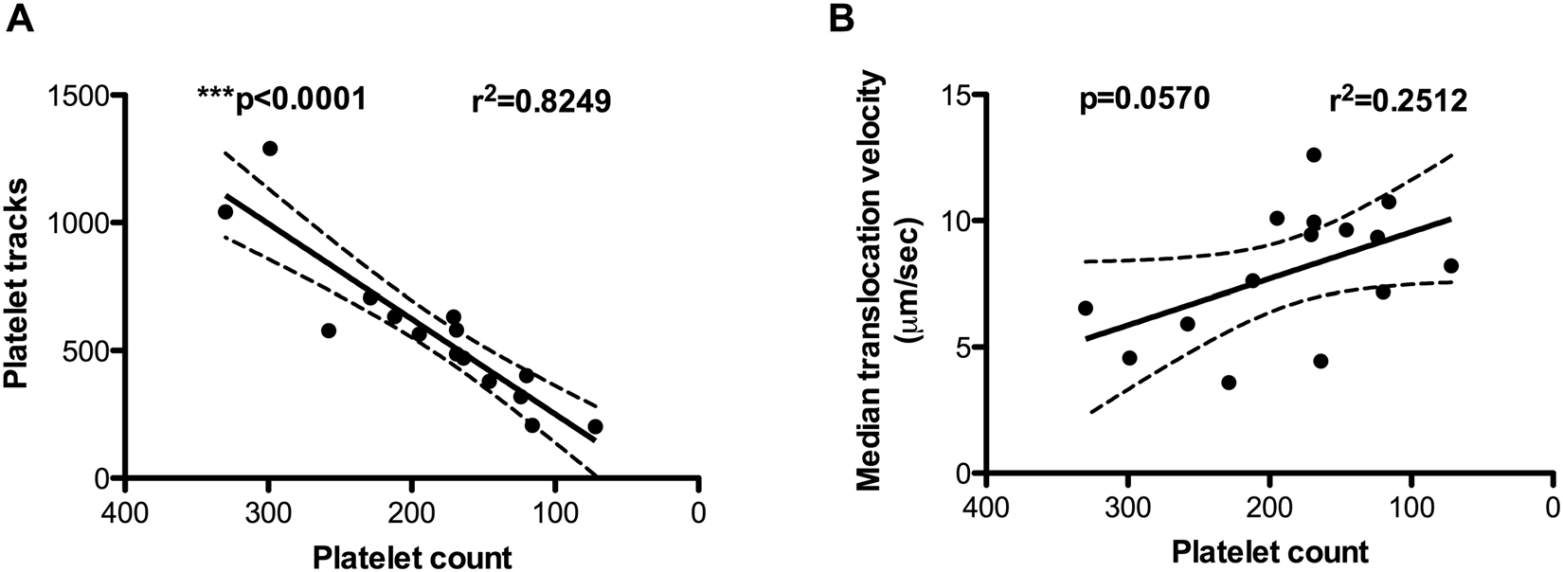
Platelet count has a significant impact on the number of platelets interacting with VWF but not on the translocation speeds. Platelets isolated from whole blood of 5 healthy control donors were diluted in platelet-poor plasma and reconstituted back into RBCs. A simple linear regression model was used to test the slopes between the platelet count and measured platelet interactions with VWF. The best-fit lines with a 95% confidence band are shown in the graph.

As pregnancy was associated with a significant reduction in platelet count compared to non-pregnant controls, we set out to characterise the relationship between platelet count and platelet interactions with VWF in the DPFA. Platelet counts from 5 healthy non-pregnant controls were manipulated to mimic a 2-fold and 3-fold haemodilution factor for each donor. There was a concomitant reduction in platelet interactions with VWF as platelet count decreased (***p < 0.0001; *r* ^2^ = 0.8249, linear regression analysis) (Fig. 2A). In marked contrast, platelet count had no effect on the speeds at which platelets translocated on VWF, indicating that the reduction in translocation speeds observed in our pregnant cohort are due to intrinsic changes in platelet function, not a result of a decrease in platelet count (*p* = 0.0570 *r* ^2^ = 0.2512, linear regression analysis, Fig. 2B).

#### Reducing HCT reduces dynamic platelet interaction with VWF

**Figure 3:**
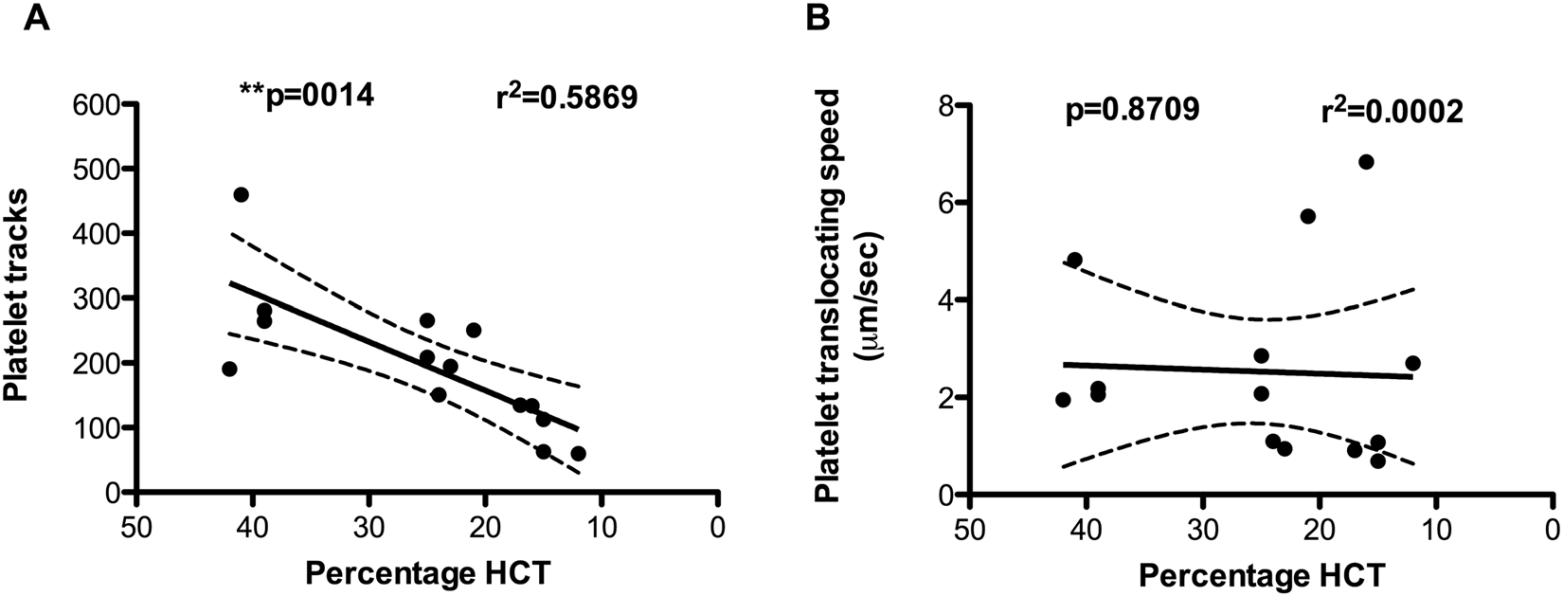
Decreasing haematocrit significantly reduces platelet interactions with VWF but has no effect on platelet translocation speeds. RBCs isolated from whole blood of 5 donors were diluted in platelet-poor plasma to mimic a 2-fold or 3-fold reduction in HCT, then reconstituted back into platelet-rich plasma. The blood was then perfused over VWF at arterial shear rates and measured using the dynamic platelet function assay. HCT correlated strongly with the extent of platelet interactions with VWF but not with the speed of platelet motion across the VWF surface. A simple linear regression model was used to test the slopes between the HCT and measured platelet interactions with VWF. The best-fit lines with a 95% confidence band are shown in the graph.

Haemodilution gave rise to a significant reduction in HCT within our cohort in their third trimester of pregnancy. To determine if the reduction in HCT had an impact on dynamic platelet interactions with VWF, we artificially reduced the HCT levels in 5 non-pregnant controls to represent a dilution of 33% and 50%. Linear regression analysis demonstrated that as haematocrit levels were reduced, so too were platelet interactions with the VWF surface of our flow chamber (**p < 0.0014; r^2^ = 0.5869, Fig. 3A). Platelet interactions with the chamber wall are driven in part by the process of margination, wherein the red blood cells (RBCs) force the smaller platelets to the periphery of the flowing blood stream^16^. There was no change in the speeds that platelet’s translocated across the surface as HCT was reduced (*p* = 0.8709; *r* ^2^ = 0.0002, Fig. 3B).

## Discussion

The results of the present investigation demonstrate that platelet function changes during healthy pregnancy. In particular, the extent of platelet interactions with VWF decreases. A significant decrease in the number of platelets translocating across and stably adhering to VWF is accompanied by a reduction in the speed of translocation (Table 2). These changes in platelet function are not just a consequence of haemodilution but due to intrinsic changes in platelet function.

The normal haemodilution associated with pregnancy is accompanied by what would appear to be a suboptimal increase in the number of RBCs in circulation: the percentage increase in blood volume exceeds the percentage increase in the number of RBCs^17^. It has been suggested that platelet count is reduced in pregnancy due to increased platelet turnover^18^. Indeed, our results for *ex*-*vivo* haemodilution of blood from non-pregnant donors show that reductions in either platelet count or HCT diminish the extent of platelet interactions with VWF.

The effects of HCT on platelet interactions with VWF have been noted previously. Chen *et al*.^19^ reported that the number of platelets that adhered to VWF is highly regulated by HCT and flow rate. As HCT and flow rate increased, driving platelets towards the vessel wall, there was a concomitant increase in platelet adhesion to VWF^19^. Our group more recently verified the importance of HCT and flow rate in determining initial platelet adhesion to VWF using Stokes flow simulations and microfluidic flow experiments^16^. At arterial shear flow (1500 s^−1^), a higher HCT was associated with greater numbers of platelet/RBC collisions. Platelet collisions with RBCs reduced the time needed for platelets to migrate through the cell-free layer towards the wall of the vessel. As more platelets presented at the vessel wall, the number of platelet interactions with VWF also increased^16^.

Haemodilution is a likely cause for the reduced numbers of platelets interacting with VWF in pregnancy. However, haemodilution cannot account for reduced platelet translocation speeds on VWF during pregnancy, a reduction we found to be independent of both platelet count and HCT. These reduced translocation speeds may result from an intrinsic change in the function of platelets that occurs uniquely during pregnancy, consistent with the enhanced platelet reactivity commonly observed in late pregnancy^3–5, 20–22^. The transient tethering of platelets to exposed VWF via the action of GPIbα is necessary to slow translocating platelets in the flowing blood stream as a prelude to stable (non-translocating) adhesion; thus, GPIbα-VWF interactions are a key determinant of translocation velocity. The slower translocation speeds observed in our pregnant cohort may be indicative, therefore, of enhanced activity of GPIbα.

In this study, we did not measure relative surface expression of GPIb or other functional parameters like CD62P surface expression (α-granule degranulation), CD63 (dense granule degranulation) and PAC-1 binding but these are likely play a role in our observations that platelet function under dynamic conditions change during pregnancy. Holthe *et al*. using flow cytometry analysis examined expression of CD61 (the Integrin beta 3 of GPIIb/IIIa complex), CD42a (GPIX of the GPIb-IX-V complex), CD62P (P-selectin), CD63 and PAC-1 binding in pre-eclamptic, normotensive pregnant and non-pregnant women^23^. They found that basal expression levels of GPIb, GPIIb/IIIa, P-selectin and PAC-1 did not differ between pregnant and non-pregnant women. Interestingly, they found that CD63 expression was increased in pregnancy at basal levels reflecting a higher level of platelet activation. This increase in CD63 associated with pregnancy has been reported previously^3^. *In*-*vitro* stimulation of platelets from pregnant and non-pregnant women with high dose Adenosine Diphosphate (ADP) demonstrated increased CD62P response in pregnancy. ADP and Thrombin receptor-activating peptide (TRAP) stimulation of platelets also resulted in increases in CD63 expression demonstrating increased platelet activation in pregnancy^23^. Recently, our group published a study in neonates in which we observed an increase in platelet translocation speeds on VWF in preterm neonates in comparison to their full-term counterparts^15^. We postulated that increased platelet translocation speed in preterms might relate to the expression of P-selectin from the alpha granules of platelets during activation. P-selectin binds to VWF and its higher expression following platelet activation in pregnancy^23^ may effect the speeds at which platelets translocate on VWF.

Using conventional platelet aggregation assays, we have also previously shown that platelet aggregation responses induced by collagen and arachidonic acid increase as pregnancy progresses^22^. This is coupled with an increase in spontaneous platelet aggregation from the 1^st^ to 3^rd^ trimester^21^. This suggests a general enhanced platelet reactivity which would reduce the time to activate the GPIIb/IIIa receptor on the platelet, which is responsible for stable platelet adhesion to VWF^11^. It is not surprising, perhaps, to find evidence that both types of receptors critical to platelet interactions are implicated as means of compensation for the haemodilution associated with pregnancy.

Valéra *et al*. recently demonstrated a reduction in overall percentage platelet surface coverage and thrombus formation on a collagen-coated surface in late pregnancy^24^. The authors used a microfluidic device to compare platelet function in pregnant females and healthy controls under arterial (1,500 s^−1^) and pathological (4,000 s^−1^) flow rates over a 2-minute time period. Platelets from pregnant patients also exhibited less interaction with immobilised fibrinogen under arterial shear. Though the mechanisms behind these findings were not explored, the authors suggested that this reduction in platelet adhesion to collagen under flow conditions might be related to oestrogen effect in pregnancy. The authors also hypothesise the reduced platelet adhesion to fibrinogen may be due to competitive binding of pregnancy-specific glycoproteins (PSGs) to the platelet receptor GPIIb/IIIa, which may represent a counteractive response to the hypercoagulable state in late pregnancy^25^. However, they did not consider the possible effects of haemodilution on their assay: a reduction in platelet count will cause a reduction in the size of thrombus formed under flow, due to the availability of fewer platelets.

Using a similar microfluidic assay to that of Valéra *et al*., we examined the initial stages of platelet interactions with VWF that are linked to the activity of GPIbα such as platelet translocation speeds and stable platelet adhesion to VWF during the first 17 seconds of image acquisition under arterial shear-flow conditions. Our system allows us to measure the early stages of thrombus development, defined in this instance as the percentage surface coverage. Measuring a range of initial platelet interactions tied to the function of a number of platelet receptors, rather than focusing on a single end-point analysis such as surface coverage, may have more predictive value in identifying patients at increased risk of post-partum haemorrhage or inappropriate thrombus development.

A limitation in this study is that we only focused on the third trimester of pregnancy. Our rationale here was to minimise the inclusion of pregnancies complicated by maternal hypertension, preeclampsia or fetal growth restriction. Given the increased haemodilution that occurs as pregnancy advances, the physiological reduction in HCT in addition to pregnancy-related changes is likely to affect platelet function at varying stages throughout pregnancy. Serial assessments of platelet parameters across all three trimesters of pregnancy is required, with particular emphasis on platelet function changes occurring during the first and second wave of placental trophoblastic invasion.

In summary, we demonstrate that healthy pregnancy results in a decrease in multiple platelet interactions with VWF. These decreases in platelet interactions are likely due to reduced numbers of platelets and levels of HCT, which occur naturally during pregnancy. For the first time, we show that platelet translocation speeds are reduced in pregnancy and are independent of haemodilution, reflecting an intrinsic change in the function of the platelet that occurs uniquely in pregnancy.

## Methods

### Ethics statement

Ethical approval for the study was obtained from the Medical Research Ethics Committee of the Royal College of Surgeons in Ireland and the Research Ethics Committee at the Rotunda Hospital, Dublin, Ireland. All participants were informed of the nature of the study and written consent was obtained from all donors prior to recruitment. All blood samples were collected in accordance with the Declaration of Helsinki.

### Study participants

All study participants were recruited from a leading Dublin maternity hospital. All assays were carried out on fresh whole blood within 60 minutes of blood draw. To fulfil the inclusion criteria, recruitment was restricted to healthy third trimester singleton pregnancies (>24 weeks’ gestational age) and healthy non-pregnant controls with no previous history of any major disease and free from any medication such as statins, anti-hypertensive medication, antiplatelet agents such as aspirin, or anti-inflammatory medications such ibuprofen, at least 12 days prior to blood draw. Pregnant patients were excluded from the study if they had diabetes mellitus, a diagnosis of fetal aneuploidy or structural fetal malformation. The third trimester was chosen as the gestational age in order to minimise the inclusion of pregnancies complicated by maternal hypertension, preeclampsia (PET) or fetal growth restriction. In addition, all pregnancies were followed by to ensure normal clinical outcome and birth weight at delivery. Following these criteria, a total of seven pregnancies were excluded after initial recruitment (subsequent diagnosis of either growth restriction after delivery (n = 2), PET (n = 2), gestational thrombocytopenia (n = 2) and obstetric cholestasis (n = 1)). In total the study included 21 healthy third trimester pregnancies and 21 matched non-pregnant controls.

### Preparation of blood

Venous blood was drawn from the antecubital vein using a 19-gauge butterfly needle connected to a sterile polypropylene syringe. Blood was drawn into 3.2% (w/v) trisodium citrate anticoagulant (1:9 volume of citrate to blood, final citrate concentration of 0.32%). Blood samples were kept at room temperature with gentle rocking and used within 1 hour of phlebotomy. Whole blood cell counts were recorded for each donor, using a Sysmex-KX21N haematology analyser (Kobe, Japan).

### Dynamic Platelet Function Assay

The Dynamic Platelet Function Assay (DPFA) was performed as previously described^13–15^. Briefly, custom parallel plate perfusion chambers were coated overnight with 100 μg/ml VWF, washed with PBS and blocked with 1% BSA for 1 hour prior to use. Whole blood was labelled with 1 μM DiOC6 for 5 minutes at 37 °C prior to perfusion through the chamber at an arterial rate of shear (1500 s^−1^). Platelet translocation behaviour was recorded using real-time video microscopy at a frame rate of 30 frames per second. Image stacks were analysed by a custom designed and validated software package^13^. The assay measurements obtained from this analysis include the number of platelets that interact with the VWF surface (Platelet tracks), the number of platelets that translocate over VWF (Translocating platelets), the average speed at which platelet translocation occurs (Platelet translocation speed), the number of platelets that stably adhere to the VWF-coated surface (Static platelets), and percent surface coverage on the final frame (Percentage of platelet surface coverage).

### Manipulation of Haematocrit and Platelet Count

Full blood counts were obtained for each blood sample using a Sysmex KX21 N hematology analyser. Blood was separated into platelet rich plasma (PRP) and packed red cells by centrifugation at 170 g for 10 minutes. To examine the effect of HCT on platelet translocating behaviour, the packed red cells were diluted by 33% or 50% by the addition of platelet poor plasma and reconstituted with the PRP fraction prior to perfusion through the flow chamber. For studies on platelet count, PRP was diluted as required with platelet poor plasma, reconstituted with the packed red cell fraction and perfused through the chambers as described.

### Statistical analysis

Blood samples were run in triplicate and the mean value of the 3 measurements was used to determine differences between our groups of interest. Normality was assessed using a D’Agostino-and-Pearson omnibus normality test. Based on the results of the D’Agostino-and-Pearson omnibus normality test, unequal variances were determined for the measured assay parameters and therefore a two-tailed unpaired Welch’s t-test was used where appropriate to elucidate differences in platelet trajectory profiles in our groups of interest. Numerical analysis was done with GraphPad Prism 6 software (GraphPad Software, Inc.). All data are presented as mean + 95% confidence interval unless otherwise stated.

## References

[1] Uchikova EH, Ledjev (2005). Changes in haemostasis during normal pregnancy. Eur J Obstet Gynecol Reprod Biol.

[2] Nicolini U (1994). Maternal and fetal platelet activation in normal pregnancy. Obstet Gynecol.

[3] Janes SL, Goodall AH (1994). Flow cytometric detection of circulating activated platelets and platelet hyper-responsiveness in pre-eclampsia and pregnancy. Clin Sci (Lond).

[4] Morrison R, Crawford J, MacPherson M, Heptinstall S (1985). Platelet behaviour in normal pregnancy, pregnancy complicated by essential hypertension and pregnancy-induced hypertension. Thromb Haemost.

[5] Sheu JR (2002). Mechanisms involved in agonist-induced hyperaggregability of platelets from normal pregnancy. J Biomed Sci.

[6] Gatti L (1994). Hemostatic parameters and platelet activation by flow-cytometry in normal pregnancy: a longitudinal study. Int J Clin Lab Res.

[7] Harlow FH (2002). Platelet activation in the hypertensive disorders of pregnancy. Am J Obstet Gynecol.

[8] Can MM (2010). Whole blood platelet aggregation failed to detect differences between preeclampsia and normal pregnancy. Platelets.

[9] Ruggeri ZM, Mendolicchio GL (2007). Adhesion mechanisms in platelet function. Circ Res.

[10] Savage B, Almus-Jacobs F, Ruggeri ZM (1998). Specific synergy of multiple substrate-receptor interactions in platelet thrombus formation under flow. Cell.

[11] Savage B, Saldivar E, Ruggeri ZM (1996). Initiation of platelet adhesion by arrest onto fibrinogen or translocation on von Willebrand factor. Cell.

[12] Kent NJ (2010). Microfluidic device to study arterial shear-mediated platelet-surface interactions in whole blood: reduced sample volumes and well-characterised protein surfaces. Biomed Microdevices.

[13] Ralph, A. *et al*. Computational Tracking of Shear-Mediated Platelet Interactions with von Willebrand Factor. *Cardiovasc Eng Technol*, doi:10.1007/s13239-016-0282-x (2016). 10.1007/s13239-016-0282-x 27743349

[14] Cowman J (2015). Age-related changes in platelet function are more profound in women than in men. Sci Rep.

[15] Cowman, J. *et al*. Dynamic platelet function on von Willebrand factor is different in preterm neonates compared with full-term neonates: changes in neonatal platelet function. *J Thromb Haemost*, doi:10.1111/jth.13414 (2016). 10.1111/jth.13414 27416003

[16] Fitzgibbon S, Cowman J, Ricco AJ, Kenny D, Shaqfeh ES (2015). Examining platelet adhesion via Stokes flow simulations and microfluidic experiments. Soft Matter.

[17] Faupel-Badger JM, Hsieh CC, Troisi R, Lagiou P, Potischman N (2007). Plasma volume expansion in pregnancy: implications for biomarkers in population studies. Cancer Epidemiol Biomarkers Prev.

[18] McCrae KR (2010). Thrombocytopenia in pregnancy. Hematology Am Soc Hematol Educ Program.

[19] Chen H (2013). Hematocrit and flow rate regulate the adhesion of platelets to von Willebrand factor. Biomicrofluidics.

[20] Juan P, Stefano G, Antonella S, Albana C (2011). Platelets in pregnancy. J Prenat Med.

[21] Burke N (2016). Reduced spontaneous platelet aggregation: a novel risk factor for adverse pregnancy outcome. Eur J Obstet Gynecol Reprod Biol.

[22] Burke N (2013). Platelet reactivity changes significantly throughout all trimesters of pregnancy compared with the nonpregnant state: a prospective study. BJOG.

[23] Holthe MR, Staff AC, Berge LN, Lyberg T (2004). Different levels of platelet activation in preeclamptic, normotensive pregnant, and nonpregnant women. Am J Obstet Gynecol.

[24] Valera MC (2015). Platelet Adhesion and Thrombus Formation in Whole Blood at Arterial Shear Rate at the End of Pregnancy. Am J Reprod Immunol.

[25] Shanley DK (2013). Pregnancy-specific glycoproteins bind integrin alphaIIbbeta3 and inhibit the platelet-fibrinogen interaction. PLoS One.

